# *NFKB1* Gene Mutant Was Associated with Prognosis of Coronary Artery Disease and Exacerbated Endothelial Mitochondrial Fission and Dysfunction

**DOI:** 10.1155/2022/9494926

**Published:** 2022-10-22

**Authors:** Jun-Yi Luo, Fen Liu, Bin-Bin Fang, Ting Tian, Yan-Hong Li, Tong Zhang, Xiao-Mei Li, Yi-Ning Yang

**Affiliations:** ^1^Department of Cardiology, The First Affiliated Hospital of Xinjiang Medical University, Urumqi, Xinjiang, China; ^2^State Key Laboratory of Pathogenesis, Prevention and Treatment of High Incidence Diseases in Central Asia, Clinical Medical Research Institute, The First Affiliated Hospital of Xinjiang Medical University, Urumqi, China; ^3^Department of Medical Science Examination Center, The First Affiliated Hospital of Xinjiang Medical University, Urumqi, China; ^4^People's Hospital of Xinjiang Uygur Autonomous Region, Urumqi, China

## Abstract

Endothelial apoptosis is the core pathological change in atherosclerotic cardiovascular disease, including coronary artery disease (CAD). Determining the molecular mechanisms underlying endothelial apoptosis is important. Nuclear factor kappa B (NF-*κ*B) is a crucial transcription factor for controlling apoptosis. Our previous study demonstrated that the -94 ATTG ins/del mutant in the promoter of *NFKB1* gene (rs28362491) is a risk factor for CAD. In the present study, we found that *NFKB1* rs28362491 polymorphism was positively associated with increased major adverse cardiac and cerebrovascular events (MACCEs) in CAD patients. After adjusting for confounding factors including age, smoking, hypertension, glucose, and low-density lipoprotein cholesterol, the mutant DD genotype was an independent predictor of MACCEs (OR = 2.578, 95%CI = 1.64–4.05, *P* = 0.003). The *in vitro* study showed that mutant human umbilical vein endothelial cells (DD-mutant HUVECs) were more susceptible to high-glucose/palmitate-induced apoptosis, which was accompanied by decreased p50 expression and increased expression of cleaved caspase-3, Cytochrome c, and phospho-p65 (*P* < 0.05). The mitochondrial membrane potential was significantly lower, while increasing levels of mtROS and more opening of the mPTP were observed in DD-mutant HUVECs (*P* < 0.05). Furthermore, the percentage of cells with fragmented or spherical mitochondria was significantly higher in DD-mutant HUVECs than in wild-type cells (genotype II HUVECs) (*P* < 0.05). In addition, after stimulation with high glucose/palmitate, the *NFKB1* gene mutant significantly increased the expression of Drp1, which indicated that the *NFKB1* gene mutant affected the expression of mitochondrial morphology-related proteins, leading to excessive mitochondrial fission. In conclusion, the mutant DD genotype of the *NFKB1* gene was an independent predictor of worse long-term prognosis for CAD patients. DD-mutant HUVECs exhibited abnormal activation of the NF-*κ*B pathway and increased Drp1 expression, which caused excessive mitochondrial fission and dysfunction, ultimately leading to increased apoptosis.

## 1. Introduction

Coronary artery disease (CAD) is the most common type of cardiovascular disease (CVD). Although lipid-lowering drugs, interventional therapies, and other conventional treatments have been widely used to reduce its threat to human health, CAD remains the primary cause of morbidity and mortality worldwide [1]. In addition, the prognosis of clinical CAD patients is still not optimistic. Hence, it is of great importance to seek potential prognostic biomarkers to identify CAD patients with high risk early, support clinical behaviour, and predict patient prognosis.

Atherosclerotic lesion formation, progression, and plaque rupture are the underlying causes of CVD [2]. However, the pathogenic mechanisms remain poorly understood. Endothelial apoptosis is a major and early step in the pathological progression of atherosclerosis (AS) [3]. Nuclear factor kappa B (NF-*κ*B) is an essential transcription factor in endothelial dysfunction and apoptosis [4]. NF-*κ*B is a dimer composed of p65 and p50 subunits. The p65 subunit carries a transcriptional activation domain and is thought to act as a transcriptional activator. In contrast, the p50 subunit lacks a transcriptional activation domain; it is considered as transcriptional repressor [5–7].

The human p50 subunit is encoded by the *NFKB1* gene, which is located on chromosome 4q24. Previous studies reported that -94 ATTG deletion mutant (DD genotype) in promoter of the *NFKB1* gene (rs28362491) was associated with inflammatory diseases, including CAD [8–10], inflammatory bowel disease [11], and ulcerative colitis [12, 13]. In our previous study, we found that the *NFKB1* mutant DD genotype was a risk factor for CAD in the Chinese Han population. The *NFKB1* gene DD mutant decreased the expression and translocation of p50 in endothelial cells, which led to increased sensitivity to oxidative stress-induced apoptosis [14]. However, whether mutations in the *NFKB1* gene DD mutant affect the long-term outcomes of CAD patients and precisely how the *NFKB1* gene influences endothelial apoptosis are unclear. Based on our previous study, in this study, we investigated the incidence of major adverse cardiac and cerebrovascular events (MACCEs) among CAD patients carrying different genotypes of *NFKB1* gene. Next, we also cultured wild-type and DD mutant human umbilical vein endothelial cells (HUVECs) to further explore the potential mechanism of *NFKB1* gene mutation in the development and progression of CAD.

## 2. Materials and Methods

The investigation conforms to the principles outlined in the Declaration of Helsinki. The Ethics Committee of the First Affiliated Hospital of Xinjiang Medical University approved all experimental protocols. Written informed consent was obtained from all participants.

### 2.1. Study Population

CAD patients were recruited at the First Affiliated Hospital of Xinjiang Medical University from 2010 to 2018. Patients who were diagnosed with CAD by coronary angiography had at least one coronary artery stenosis with >50% reduction of the luminal diameter. Those with concomitant valvular heart disease, congenital heart disease, and/or nonischaemic cardiomyopathy were excluded. Ultimately, we recruited 785 CAD patients in our study. They received medical treatments recommended by the guidelines, such as lifestyle intervention, secondary drug prophylaxis, and/or revascularization [15, 16].

### 2.2. Genotyping

Genomic DNA was isolated from peripheral vein blood from human subjects using a TIANamp Genomic DNA Kit (Tiangen Biotech, China). The genotypes of the NFKB1 gene rs28362491 were detected by SNPscan™ typing assays with double ligation and multiplex fluorescence PCR (Cat#: G0104; Genesky Biotechnologies Inc., Shanghai, China). The probes for the identification of primer sequences were 5′-GCCTGCGTTCC CCGACCACTG-3′ and 5′-TGCTGCCTGCGTTCCCCGTCC-3′. The universal primer was ATTGGGCCCGGCAGGCGCTT.

### 2.3. Follow-Up

In the evaluation of long-term clinical outcomes, MACCEs were considered the end points, including cardiac and noncardiac death, nonfatal acute myocardial infarction (AMI), unplanned revascularization (new percutaneous coronary intervention (PCI) or bypass cardiac surgery), malignant arrhythmia, development of congestive heart failure, and stroke. Participants were followed up every year after discharge through telephone communication or face-to-face interviews with the patients or their family members by a trained research cardiologist using a structured questionnaire. At last, 84 patients (10.7%) were lost to follow-up.

### 2.4. Cell Culture and Treatment

HUVECs were isolated from freshly collected neonatal umbilical cords after informed consent was obtained from the puerperae. Genomic DNA was extracted from umbilical cords for genotyping of *NFKB1* gene rs2836249 polymorphism. The method of genotyping for *NFKB1* gene rs2836249 was the same as described in the previous section on population study [14]. Cells were grown in endothelial cell medium (ECM) supplemented with 5% FBS, 1% endothelial cell growth supplement, and penicillin-streptomycin (ScienCell, CA). HUVECs were cultured in a 5% CO_2_/95% air atmosphere at 37°C. HUVECs were treated with 25 mM glucose and 250 *μ*M palmitic acid (PA) for 3 h to induce high-glucose/palmitate injury according to previous studies [17, 18].

### 2.5. Assessment of Apoptosis

After treatment with high glucose/palmitate, the cells were removed from the dishes with 0.25% trypsin-EDTA (Gibco-BRL, USA), gently washed twice with cold phosphate-buffered saline (PBS), and centrifuged at 200 × *g* for 5 minutes. The cells were stained with 100 *μ*L of Annexin-V-FLUOS labelling solution (Roche, Germany) and incubated for 15 minutes at room temperature. Then, 0.5 mL incubation buffer was added and analyzed by a flow cytometer (BD Biosciences, USA) using 488 nm excitation and a 515 nm bandpass filter for fluorescein detection and a filter > 600 nm for propidium iodine (PI) detection. Early apoptotic cells were Annexin V-FITC positive and PI negative. Cells that were in late apoptosis or already dead showed both Annexin V-FITC and PI positivity.

### 2.6. Western Blotting

Total proteins were extracted from cells using RIPA buffer and quantitated by a Pierce® BCA Protein Assay Kit (Thermo Scientific, USA). The proteins (20 *μ*g) were fractioned on 4%-12% SDS–PAGE gels (Invitrogen, USA) and transferred onto a polyvinylidene fluoride membrane (Merck, Germany). The membrane was blocked with 5% skim milk for 1 h at room temperature, incubated with the primary antibody overnight at 4°C, washed with tris-buffered saline Tween 20 (TBST) 3 times, incubated with the secondary antibody (1 : 5000) for 1 h at room temperature, and washed with TBST 3 times. The protein bands were detected with SuperSignal West Pico-enhanced chemiluminescent solution (Thermo Scientific, USA). The integrated density of immune blots was determined by ImageJ software (National Institute of Mental Health, USA). Primary antibodies against p50 (1 : 1000), p65 (1 : 1000), cleaved caspase-3 (1 : 1000), Drp1 (1 : 1000), mitofusion-2 (1 : 1000), Cyto c (1 : 1000), and *β*-actin (1 : 1000) and secondary antibodies were purchased from Cell Signaling Technology (CST, USA). Primary Opa1 (1 : 1000) antibodies were obtained from Abcam (Cambridge, UK).

### 2.7. Assessment of Mitochondrial Morphology and Function

Visualization of mitochondria within cells was performed with MitoTracker Green FM (Invitrogen, USA), which stains mitochondria regardless of mitochondrial membrane potential. Live cells were grown in confocal dishes and treated with glucose and palmitate as described above. After stimulation, cells were washed with warm buffer and incubated with 200 nM MitoTracker Green at 37°C for 30 minutes. Cells were gently washed with PBS three times. MitoTracker Green was excited using the 488 nm laser line, and fluorescence was measured using a 495–523 nm bandpass filter by confocal microscopy equipped with a ×63 oil immersion objective.

The mitochondrial membrane potential was assessed using a JC-1 probe (Invitrogen, USA). Following glucose and PA treatment, HUVECs were supplemented with 0.5 *μ*g/mL JC-1 (30 minutes at 37°C) and returned to ECM for microscopy. Green monomeric JC-1 and red aggregated JC-1 were detected at emission wavelengths of 530 nm and 590 nm, respectively. Quantitation of red and green fluorescent signals reflects whether mitochondria are damaged.

Mitochondrial ROS generation was measured using a MitoSOX Red probe (Invitrogen, USA), which is a probe that specifically reacts with mitochondrial superoxide anion, according to the manufacturer's instructions. Briefly, cells were washed with serum-free DMEM media. Then, the cells were stained with serum-free media containing 5 *μ*M MitoSOX Red probe and incubated at 37°C in the dark for 30 min. Subsequently, the cell pellets were washed three times with PBS and returned to ECM for microscopy.

In cell apoptosis, mPTP opening plays a major role, and the Ca_2_^+^-induced mitochondrial swelling assay can be used to determine mPTP opening by a Cell mPTP Assay Kit (Beyotime, China). It contains assay buffer, calcein-AM (100x), CoCl_2_ (100x), ionomycin (200x), and solubilizing agent (100x). According to the manufacturer's instructions, the assay buffer contains calcium to maintain normal conditions for HUVECs over a period of time. A decrease in mitochondrial calcein-AM fluorescence reflects the opening of the mPTP. First, the culture medium was discarded, and the cells were rinsed gently with PBS. Subsequently, the cells were incubated with the staining working solution at 37°C in the dark for 30 min. Finally, the cells were washed 3 times with PBS and returned to the assay buffer. A laser scanning confocal microscope was used for the analysis.

### 2.8. Statistical Analysis

All computations were performed with SPSS software 22.0 (SPSS Inc., Chicago, IL). For CAD patient data, categorical variables are presented as *n* (%), and continuous variables are presented as the mean ± standard deviation. The chi-square test and independent-sample Student's *t* test were used to analyze categorical variables and continuous variables, respectively. Survival curves were estimated by the Kaplan–Meier (KM) method. The association between different genotypes and other risk factors and MACCEs was estimated by Cox regression analyses with odds ratios (ORs) and 95% confidence intervals (CIs). For the cell experiments, the data of each figure consist of a representative experiment of the independent set of experiments. All acquired images by microscopy were processed and analyzed by ImageJ (National Institutes of Health, Bethesda, USA) open source software. Statistical analyses were performed using Prism 6 (GraphPad Software, USA). Data are expressed as the mean ± standard error of the mean (SEM). Statistical significance was determined by comparing the means between groups by two-way ANOVA. *P* values < 0.05 (∗) were deemed statistically significant.

## 3. Results

### 3.1. *NFKB*1 Gene DD Mutant Was a Risk Factor for MACCE Incidence in CAD Patients

The clinical characteristics of CAD patients with or without MACCEs are summarized in Table 1. Among the 701 participants who had completed follow-up during a median follow-up period of 40.3 (18.1–71.8) months, 177 subjects (25.2%) had MACCE, including 31 cardiac deaths, 17 noncardiac deaths, 22 had nonfatal AMI, 52 had unplanned revascularization, 7 had malignant arrhythmia, 36 had cardiac failure, and 12 had stroke.

During the follow-up, *NFKB1* gene DD mutant was more common in CAD patients who underwent MACCE (27.1%) than in those who did not suffer MACCE (13.9%) (*P* < 0.001). Similarly, participants carrying DD genotype had a significantly higher unplanned revascularization incidence (32.7%) (*P* = 0.001). The detailed data of MACCE incidence are shown in Table 2.

**Figure 1:**
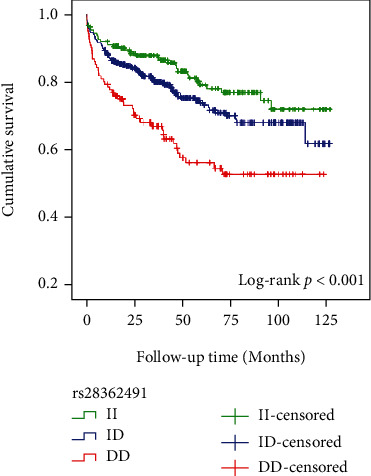
Kaplan-Meier curves for MACCEs according to different genotypes of *NFKB1* gene rs28362491.

As shown in Figure 1, Kaplan-Meier survival curves indicated that those with DD genotype had a higher risk of MACCEs than those with the ID or II genotype (*P* < 0.001). After adjustment for age, smoking, DM, hypertension, glucose, and LDL-C, Cox regression analysis showed that the *NFKB1* DD mutant was associated with a higher incidence of MACCE (DD versus II: adjusted OR = 2.578, 95%CI = 1.64–4.05, *P* = 0.003). Traditional risk factors, such as increased age and high levels of glucose and LDL-C, were also independent predictors of MACCEs (all *P* < 0.05) (Table 3).

### 3.2. HUVECs with *NFKB1* Gene DD Mutant Were More Vulnerable to High-Glucose/Palmitate-Induced Apoptosis

**Figure 2:**
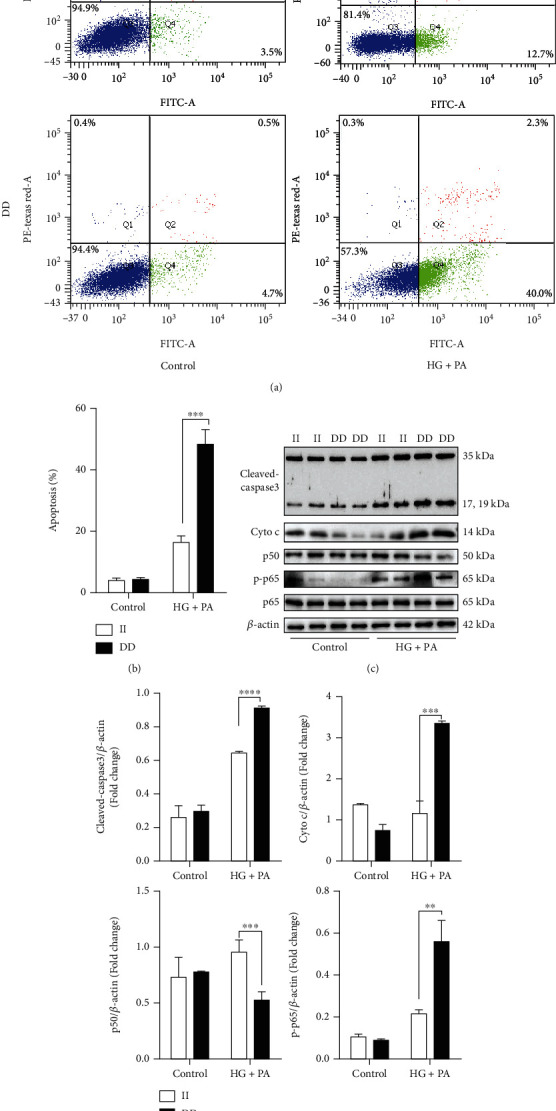
HUVECs with the *NFKB1* gene DD mutant were more vulnerable to high-glucose/palmitate-induced apoptosi*s*. (a) FACS analysis of Annexin V-FITC/PI-labeled HUVECs was performed to quantify apoptosis. Cells in the lower right quadrant indicated Annexin-positive/PI-negative, early apoptotic cells. (b) The percentage of cells undergoing early apoptosis in comparison with the respective control after 3 h high-glucose/palmitate treatment (*n* = 3). (c) Representative western blot bands of apoptotic-related proteins after treatment with high glucose/palmitate in II genotype and DD mutant HUVECs. (d) The bar graphs showed the relative levels of cleaved-capase3, p65, phospho-p65, and p50, respectively (*n* = 4). Values are means ± SEM. ^∗∗^*P* < 0.01, ^∗∗∗^*P* < 0.001, and ^∗∗∗∗^*P* < 0.0001.

Following the above findings, high levels of glucose and palmitate were used to induce HUVEC injury to mimic the in vivo environment that occurs in the early stages of AS plaque formation. After treatment with high glucose/palmitate, the apoptosis rate was 30% higher in DD-mutant HUVECs than in II genotype HUVECs (Figures 2(a) and 2(b)). Western blot analysis showed that cleaved caspase-3, Cytochrome c (Cyto c), and phospho-p65 protein expression was significantly higher in DD-mutant HUVECs than in II genotype HUVECs after the induction of high glucose/palmitate (all *P* < 0.05), while the expression of p50 was significantly lower in DD mutant cells (*P* < 0.001) (Figures 2(c) and 2(d)). These results indicated that *NFKB1* gene DD mutant could increase the expression of proapoptotic molecules by aberrantly activating the NF-*κ*B signaling pathway under the induction of high glucose/palmitate.

### 3.3. Mitochondria Were More Sensitive to High-Glucose/Palmitate Damage in *NFKB1* Gene DD Mutant Cells

**Figure 3:**
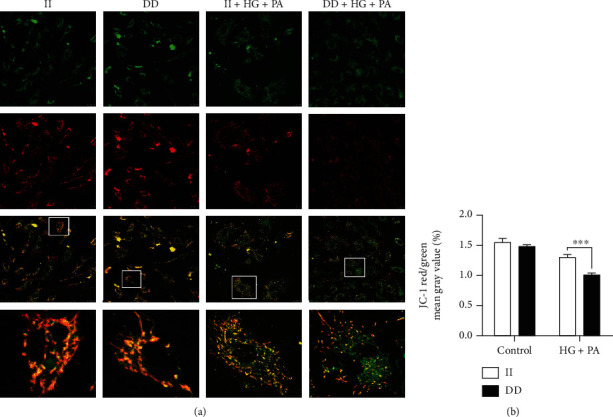
Mitochondrial membrane potential decreased more significantly in *NFKB1* DD-mutant cells. (a) Representative confocal photomicrographs stained with JC-1. (b) JC-1 red/green means gray value. HUVECs were treated with 25 mM glucose and 250 *μ*M palmitic acid (PA) for 3 hours. Data represents the mean ± SEM of at least three independent cell culture preparations (more than 10 visual fields per group). ^∗∗∗^*P* < 0.001.

The decrease in mitochondrial membrane potential was a hallmark of early cell apoptosis. The enhanced green fluorescence (depolarized mitochondria) of JC-1 indicated lower mitochondrial membrane potential, while the red colour indicated higher mitochondrial membrane potential (healthy mitochondria). Consequently, a decreased red/green ratio is an indicator of mitochondrial membrane depolarization. As shown in Figure 3, after the induction of high glucose/palmitate, the red/green ratio significantly decreased in DD-mutant HUVECs, indicative of severely impaired mitochondrial function in DD-mutant HUVECs compared with II genotype cells (*P* < 0.001).

**Figure 4:**
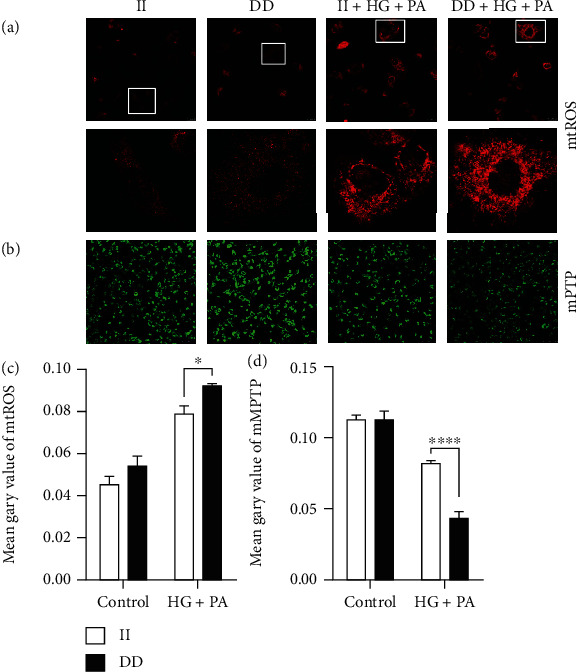
Mitochondria were more sensitive to high-glucose/palmitate damage in *NFKB1* DD-mutant cells. (a, b) Representative confocal photomicrographs stained with MitoSOX Red and mPTP. (c, d) Mean fluorescent intensities of MitoSOX and mPTP per cell. HUVECs were treated with 25 mM glucose and 250 *μ*M palmitic acid (PA) for 3 hours. Data represents the mean ± SEM of at least three independent cell culture preparations (more than 10 visual fields per group). ^∗^*P* < 0.05 and ^∗∗∗^*P* < 0.001.

To further confirm the effect of *NFKB1* DD mutant on mitochondrial function, we then evaluated the ROS level special in mitochondrial (mtROS) and mitochondrial permeability transition pore (mPTP) opening. As demonstrated by fluorescence images, a greater increase in the production of mtROS was observed in DD-mutant HUVECs than in II genotype cells after treatment with high glucose/palmitate (Figures 4(a) and 4(c)). In addition, the fluorescence images also demonstrated that the mean fluorescence intensities (green) were significantly lower in DD-mutant HUVECs under the induction of high glucose/palmitate, indicating that *NFKB1* DD mutant promoted mPTP opening, leading to greater mitochondrial dysfunction (Figures 4(b) and 4(d)).

### 3.4. *NFKB1* Gene DD Mutant Caused Aberrant Mitochondrial Fission

**Figure 5:**
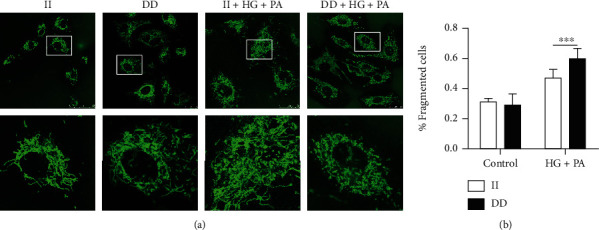
The *NFKB1* gene DD mutant caused aberrant mitochondria fission. All cells were stained with MitoTracker Green FM and then analyzed by ImageJ. (a) Representative confocal photomicrographs showing mitochondrial morphology imaged by laser scanning confocal microscopy. (b) The percentage of cells with fragmented or spherical mitochondria in each group. HUVECs were treated with 25 mM glucose and 250 *μ*M palmitic acid (PA) for 3 hours. Data represents the mean ± SEM of at least three independent cell culture preparations (more than 10 visual fields per group). ^∗^*P* < 0.05.

Mitochondrial dysfunction is suggested to serve as a key pathophysiological determinant of endothelial apoptosis. To assess the effect of *NFKB1* DD mutant on mitochondrial morphology and network organization, we assessed mitochondrial morphology in both mutant and wild-type HUVECs with a MitoTracker probe. Under normal culture conditions, the mitochondria diffused throughout the cell, with an oval or rod-like shape and normal structure. After treatment with high glucose/palmitate, the mitochondria were mostly shorter and dot-shaped (fragmented or spherical mitochondria). In DD-mutant HUVECs, the percentage of cells with fragmented or spherical mitochondria was significantly higher in DD-mutant HUVECs than in wild-type cells (*P* < 0.05, Figure 5).

### 3.5. *NFKB1* Gene DD Mutant Increased the Expression of the Mitochondrial Fission Factor Drp1

**Figure 6:**
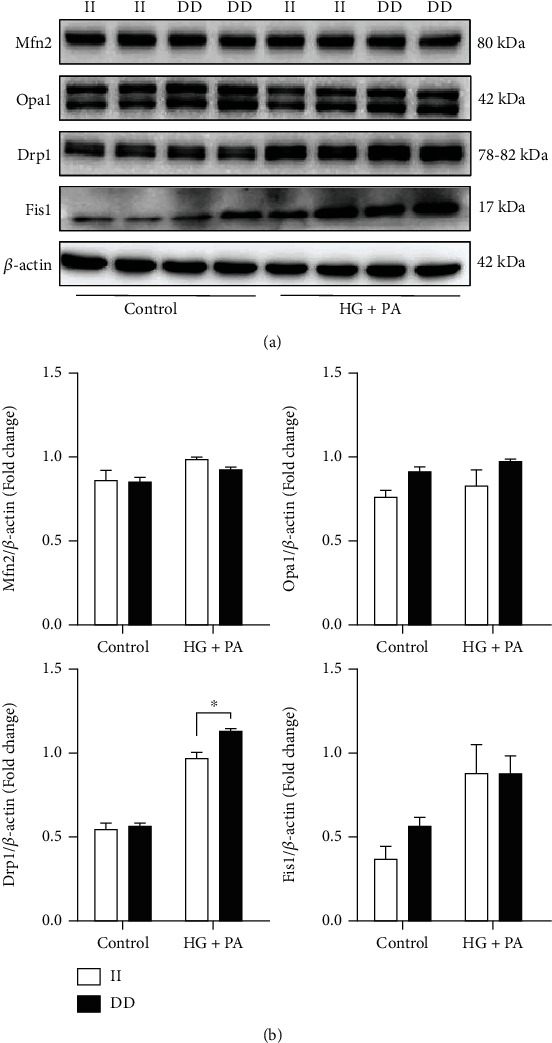
The *NFKB1* gene DD mutant increased the expression of mitochondrial fission-related proteins. (a) Expression of mitochondrial morphology-related proteins by western blot after treatment with high-glucose/palmitate. (b) The bar graphs showed the relative levels of mitochondrial morphology-related proteins (*n* = 4). HUVECs were treated with 25 mM glucose and 250 *μ*M palmitic acid (PA) for 3 hours. Values are means ± SEM. ^∗^*P* < 0.05.

To further explore how the *NFKB1* gene influences mitochondrial morphology, we measured the expression of mitochondrial morphology-related proteins by western blot. As shown in Figure 6, after stimulation with high glucose/palmitate, *NFKB1* gene mutant significantly increased dynamic-related protein 1 (Drp1) expression compared with II genotype cells, which indicated that the *NFKB1* gene mutant affected the expression of mitochondrial morphology-related proteins, leading to excessive mitochondrial fission.

## 4. Discussion

This study investigated the influence of *NFKB1* gene rs28362491 polymorphism on CAD patients' long-term prognosis and the potential mechanism underlying *NFKB1* gene mutation in the development and progression of CAD. *NFKB1* gene DD mutation was positively associated with increased MACCEs in CAD patients. After adjusting for confounding factors, *NFKB1* DD mutant genotype was an independent predictor of MACCEs. In addition, *NFKB1* gene DD mutant increased Drp1 expression, which caused aberrant mitochondrial fission and dysfunction, was the potential mechanism of increased endothelial apoptosis induced by high glucose/palmitate.

The *NFKB1* gene rs28362491 polymorphism was first reported to be a susceptible factor for ulcerative colitis in 2004 [13]. Since then, several studies, including our previous studies, have reported that *NFKB1* rs28362491 DD mutant genotype is a risk factor related to CAD among different genetic background populations [9, 10, 14, 19]. However, whether *NFKB1* rs28362491 DD mutant influences the incidence of MACCEs in CAD patients is still unclear. In the present study, we first found that *NFKB1* DD mutant was associated with a higher incidence of MACCEs than CAD patients carrying the II genotype. During the long-term follow-up, the total incidence of MACCEs in CAD patients was 25.2%. Notably, the MACCE incidence in CAD patients with the DD mutant genotype was more than 2 times higher than that in patients carrying the II genotype. Cox regression further suggested that the mutant DD genotype was an independent predictor of MACCEs. We also noted that the divergence of MACCE incidence might arise from the difference in unplanned revascularization. CAD patients with *NFKB1* DD mutant genotype were more prone to unplanned revascularization, which was approximately three times higher than patients carrying the II genotype. It is well known that unplanned revascularization is the main solution for AS plaque progression resulting in coronary stenosis [20]. From this perspective, we speculated that the *NFKB1* DD mutant gene may serve as a biomarker of AS plaque progression.

Currently, accumulating evidence has revealed that dysfunction of the vascular endothelium is an important contributor to the progression of atherosclerotic plaque progression [21, 22]. Excess apoptosis of endothelial cells induced by traditional risk factors for AS is an important event in AS development and might be a target for preventing and treating AS [21, 23, 24]. According to our results, high levels of glucose and LDL-C were independent and risk predictors of MACCEs. Hence, high levels of glucose and palmitate were used to induce HUVEC apoptosis to mimic AS development. It has been reported that endothelial dysfunction and apoptosis are the earliest and the most critical events in the onset and progression of atherosclerosis. Endothelial cells exposed to hyperglycaemia and hyperlipidaemia undergo apoptotic processes, leading to the detachment of endothelial cells from the intima. This initial denudation consequently triggers proatherosclerotic responses, resulting in the development of atherosclerosis. As we know, HUVECs differ from other venous endothelia in adult tissue, since the umbilical vein transports oxygenated and nutrient-rich blood to the fetus (arterial blood). In addition, due to easy access and high purity, HUVEC is the valuable model for the study of physiopathological processes of cardiovascular disease. Thus, in our study, primary HUVECs were isolated from neonatal umbilical cords with different genotypes of *NFKB1* gene rs28362491. At the cytological level, we found that *NFKB1* gene mutation increased apoptosis by approximately 30% in HUVECs with the DD genotype compared with cells with the II genotype. Supportively, cleaved caspase-3 was also significantly upregulated in cells with the DD genotype. Caspase-3 is a critical executioner in the downstream signaling pathway of apoptosis [25]. When activated by risk factors, caspase-3 becomes cleaved caspase-3 and initiates apoptosis [26, 27]. Therefore, cleaved caspase-3 is often used as a reliable indicator to determine the severity of apoptosis. We also noted that the *NFKB1* mutant gene markedly increased phospho-p65 expression in DD cells, indicating that the *NFKB1* mutant gene aberrantly activated the NF-*κ*B signaling pathway. Together, under high-glucose/palmitate treatment, *NFKB1* gene mutation may increase caspase-3 activity by abnormally activating the NF-*κ*B signaling pathway.

As we know, NF-*κ*B is an essential transcription factor mediated in endothelial dysfunction, apoptosis, and inflammation [28–30]. It regulates the expression of many genes, such as genes encoding cytokines (interleukin-2 (IL-2), IL-6, IL-12, tumor necrosis factor-*α* (TNF-*α*), and interferon-*γ* (IFN-*γ*)), cell adhesion molecules, and endothelial nitric oxide synthase (eNOS) [31, 32]. In our previous study, we found that IL-6 level was higher, but eNOS level was lower in peripheral circulation of CAD patients with *NFKB1* gene DD mutant compared to CAD patients carrying II genotype. Moreover, our *in vitro* study showed that oxidative stress induced lower eNOS but higher IL-6 mRNA expression in *NFKB1* gene DD mutant HUVECs than that in II genotype HUVECs [14]. For apoptosis, mainly the intrinsic pathway, it is also governed by the mitochondria function [33]. In the intrinsic pathway, mitochondria dysfunction, characterized by mPTP opening and mitochondrial membrane potential depolarization, is a pivotal regulator in controlling the release of proapoptotic proteins, such as Cyto c, which can trigger the mitochondrial apoptosis cascade [34]. The Cyto c released from mitochondria initiates the assembly of apoptosomes, activating factor 1 and caspase 9, an initiator caspase that cleaves and activates caspase-3 [35]. In the present study, after the induction of high glucose/palmitate, *NFKB1* gene mutant markedly decreased mitochondrial membrane potential and promoted the opening of the mPTP. In addition, MitoSOX staining demonstrated that the mtROS level was higher in DD mutant cells. Alnahdi et al. reported that the presence of high levels of glucose and palmitate also enhanced mitochondria dysfunction and apoptosis in HepG2 cells while suppressing its autophagy by inducing inflammatory and oxidative stress responses via activating NF-*κ*B/AMPK/mTOR pathway [36]. Shulin et al. found inhibiting the activation of NF-*κ*B may protect against mitochondrial dysfunction in acetaminophen-induced acute liver injury [37]. Therefore, combined with the above results, the *NFKB1* mutant gene may worsen mitochondrial function via affecting NF-*κ*B activity under high-glucose/palmitate treatment.

**Figure 7:**
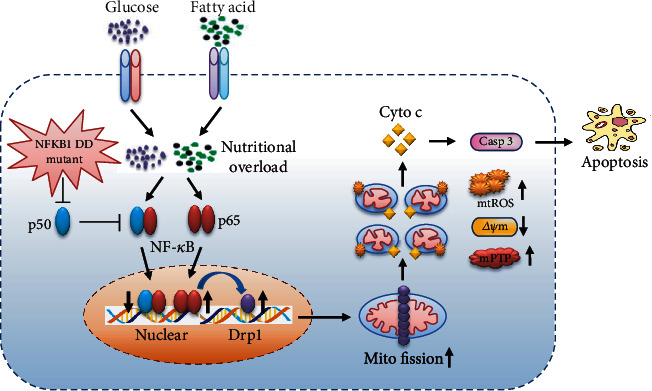
The hypothesis of *NFKB1* gene mutant exacerbated endothelial mitochondrial fission and dysfunction.

The equilibrium of mitochondrial dynamics and normal mitochondrial morphology are critical for maintaining mitochondrial function [38]. Mitochondria are dynamic organelles whose morphology is directly linked to the maintenance of their functions, and the disruption of their normal shape is a hallmark of mitochondrial dysfunction [39, 40]. Mitochondrial dynamics are regulated by the balance of fission and fusion; imbalances in fission and fusion cause mitochondrial abnormalities that lead to many diseases, including cardiovascular disease [41, 42]. Mitochondrial morphology is regulated by a series of proteins that are involved in mitochondrial fusion and fission, including fission proteins such as Drp1, fission protein 1 (Fis1), mitochondria fission factor (Mff) and mitochondria dynamics proteins of 49 and 51 kDa (MiD49, MiD51) and fusion proteins such as mitofusins (Mfn1 and Mfn2) and optic atrophy 1 (Opa1) [43]. Among these fission proteins, Drp1 is known as a crucial factor in mitochondrial fission. In the present study, we found that *NFKB1* gene mutation significantly increased the expression of Drp1 after stimulation with high glucose/palmitate. Laforge and his colleagues reported that the nonclassical NF-*κ*B pathway was involved in the regulation of mitochondrial dynamics and Opa1 expression in mouse embryonic fibroblast cells [44]. Similar to a previous study, a *NFKB1* gene mutant decreased p50 expression and led to abnormal activation of the NF-*κ*B pathway, which promoted the expression of its downstream genes, including the inflammatory factor IL-6 [14]. Karban et al. [13] and Park et al. [45] reported that mutation of *NFKB1* DD gene significantly reduced p50 subunit promoter activity in HT-29 human colonic epithelial cells and HUVECs. Furthermore, it has been reported that the p50 subunit lacks a transcriptional activation domain and is considered a transcriptional repressor [5–7]. Thus, we speculated that *NFKB1* gene mutant could increase Drp1 expression and may have caused excessive mitochondrial fission that led to mitochondrial dysfunction and HUVEC apoptosis under stimulation with high glucose/palmitate (Figure 7).

In summary, our data demonstrated that the mutant DD genotype of *NFKB1* gene was associated with worse long-term prognosis for CAD patients, and the mutant DD genotype was an independent predictor of MACCE. Furthermore, under the induction of high glucose/palmitate, mutant DD-carrying HUVECs exhibited abnormal activation of the NF-*κ*B pathway and increased Drp1 expression, which caused excessive mitochondrial fission, ultimately leading to increased apoptosis.

## Figures and Tables

**Table 1:** Demographics, clinical baseline characteristics.

	Non-MACCE (*n* = 524)	MACCE (*n* = 177)	*P* value
Age, median (year)	57.4 ± 11.5	60.8 ± 11.3	0.001
Male (*n*, %)	154 (29.4)	48 (27.1)	0.564
Smoking (*n*, %)	243 (46.4)	73 (41.2)	0.236
Drinking (*n*, %)	173 (33.0)	47 (26.6)	0.109
Diabetes (*n*, %)	135 (25.8)	49 (27.7)	0.616
Hypertension (*n*, %)	270 (51.5)	95 (53.7)	0.621
BMI (kg/m^2^)	25.7 ± 3.4	25.4 ± 3.3	0.330
SBP (mmHg)	124 ± 18	123 ± 21	0.424
DBP (mmHg)	76 ± 12	75 ± 13	0.239
TG (mmol/L)	1.9 ± 1.0	1.9 ± 1.3	0.812
TC (mmol/L)	4.3 ± 1.0	4.3 ± 1.3	0.839
LDL-C (mmol/L)	2.8 ± 0.8	3.0 ± 0.8	0.002
HDL-C (mmol/L)	1.0 ± 0.3	1.0 ± 0.3	0.181
Glucose (mmol/L)	7.4 ± 2.4	8.8 ± 3.8	<0.001
Gensini score (*n*)	53.0 ± 38.7	64.8 ± 43.9	0.001
Stents (*n*)	1.0 ± 0.7	0.9 ± 0.7	0.394

Continuous data were presented as mean ± SD. Categorical data were presented as the number (percentage). MACCE: major adverse cardiac and cerebrovascular event; BMI: body mass index; SBP: systolic blood pressure; DBP: diastolic blood pressure; TG: triglycerides; TC: total cholesterol; LDL-C: low-density lipoprotein cholesterol; HDL-C: high-density lipoprotein cholesterol.

**Table 2:** MACCE and all-cause mortality events among CAD patients carrying different genotypes of NFKB1 gene rs28362491.

	II (*n* = 213)	ID (*n* = 367)	DD (*n* = 121)	*P* value
Non-MACCE (*n*, %)	173 (33.0)	278 (53.1)	73 (13.9)	—
MACCE (*n*, %)	40 (22.6)	89 (50.3)	48 (27.1)	<0.001
All-cause mortality (*n*, %)	13 (27.1)	24 (50.0)	11 (22.9)	0.226
Cardiac mortality (*n*, %)	10 (32.3)	13 (41.9)	8 (25.8)	0.172
Noncardiac mortality (*n*, %)	3 (17.6)	11 (64.7)	3 (17.6)	0.412
Nonfatal AMI (*n*, %)	5 (22.7)	11 (50.0)	6 (27.3)	0.190
Unplanned revascularization (*n*, %)	11 (21.2)	24 (46.2)	17 (32.7)	0.001
Malignant arrhythmia (*n*, %)	1 (14.3)	5 (71.4)	1 (14.3)	0.555
Cardiac failure (*n*, %)	8 (22.0)	18 (50.0)	10 (27.8)	0.060
Stroke (*n*, %)	2 (16.7)	7 (58.3)	3 (25.0)	0.361

MACCE: major adverse cardiac and cerebrovascular event; AMI: acute myocardial infarction.

**Table 3:** Predictors of MACCE by Cox regression.

Variables	Odds ratio	95% CI	*P* value
Age	1.024	1.009–1.039	0.001
Smoking	1.155	0.831–1.605	0.392
DM	1.709	1.120–2.604	0.013
Hypertension	1.045	0.760–1.437	0.787
Glucose	1.177	1.116–1.650	<0.001
LDL-C	1.358	1.117–1.650	0.002
rs28362491 genotypes			
ID vs. II	1.341	0.896–2.007	0.154
DD vs. II	2.578	1.640–4.050	0.003

CI: confidence interval; DM: diabetes mellitus; LDL-C: low-density lipoprotein cholesterol.

## Data Availability

All of the data were presented in the main paper. The data that support the findings of this study are available on request from the corresponding author. All authors take responsibility for the integrity of the data and the accuracy of the data analysis.
